# Effectiveness of social giving on the engagement of pharmacy professionals with a computer-based education platform: a pilot randomized controlled trial

**DOI:** 10.1186/s12909-022-03310-0

**Published:** 2022-04-07

**Authors:** Rand Hussein, Rosemary Killeen, Kelly Grindrod

**Affiliations:** School of Pharmacy, University of Waterloo, Waterloo, ON Canada

**Keywords:** Pharmacy professionals, Computer-based education, Engagement, Reward

## Abstract

**Background:**

Computer-based education is gaining popularity in healthcare professional development education due to ease of distribution and flexibility. However, there are concerns regarding user engagement. This pilot study aims to: 1) assess the feasibility and acceptability of a social reward and the corresponding study design; and 2) to provide preliminary data on the impact of social reward on user engagement.

**Methods:**

A mixed method study combing a four-month pilot randomized controlled trial (RCT), surveys and interviews. The RCT was conducted using a computer-based education platform. Participants in the intervention group had access to a social reward feature, where they earned one meal for donation when completing a quiz with a passing score. Participants in the control group did not have access to this feature. Feasibility and acceptability of the social reward were assessed using surveys and telephone interviews. Feasibility of the RCT was assessed by participant recruitment and retention. User engagement was assessed by number of quizzes and modules completed.

**Results:**

A total of 30 pharmacy professionals were recruited with 15 users in each arm. Participants reported high acceptability of the intervention. The total number of quizzes completed by the intervention group was significantly higher compared to the control group (*n* = 267 quizzes Vs. *n* = 97 quizzes; *p*-value 0.023).

**Conclusion:**

The study demonstrates the feasibility and acceptability of a web-based trial with pharmacy professionals and the social reward intervention. It also shows that the social reward can improve user engagement. A future definitive RCT will explore the sustainability of the intervention.

**Supplementary Information:**

The online version contains supplementary material available at 10.1186/s12909-022-03310-0.

## Background

Computer-based education, defined as the delivery of educational content through information and communication technologies (ICT)” [1], has become a popular intervention for professional development for healthcare professionals. Computer-based education employs a wide variety of features such as traditional lectures, clinical simulations, games, and online discussion forums [2]. It offers an easily updated, widely distributable, and more flexible alternative to traditional learning, which makes it ideal for busy healthcare professionals who are expected to keep updated and maintain their competence. Moreover, computer-based education can accommodate different learning styles, allow for self-paced learning, and unlimited access to online resources [2–4]. Several studies have reported positive results with computer-based education on healthcare professionals’ knowledge compared to no intervention [5, 6]. However, this effectiveness is mediated by how engaging the computer-based education is [7]. Moreover, there are significant concerns that computer-based education maybe associated with learner isolation due to lack of face-to-face interaction and lack of accountability, leading to lower engagement and high dropout rates [1, 4, 8]. Hence, innovative approaches are needed to maximize user engagement.

Gamification, defined as the use of game elements in non-game contexts [9], is gaining in popularity as a method for enhancing user engagement and motivation, including for content directed at healthcare professionals [10–13]. In computer-based education, gamification can enhance both extrinsic and intrinsic motivation through different game elements. Common game elements are rewards, feedback, and challenges. Rewards, in particular, have shown positive results in enhancing users’ participation and engagement with online platforms [14, 15]. There are different types of rewards: monetary (e.g., payment, bonus and coupons); virtual points (e.g., points collected in the game); and social (e.g., peer recognition and compliments) [16, 17]. Studies have shown that non-monetary rewards have a more powerful impact on users’ engagement especially when they are perceived as credible, and culturally meaningful [14]. Moreover, literature has shown that pharmacists are largely motivated by social rewards [18].

Pharmacy5in5 was launched in January 2018. It is a computer-based learning platform (hosted at Pharmacy5in5.ca) aiming to help Canadian pharmacy professionals build their knowledge and skills related to pharmacy practice. In developing the platform, one of the challenges was to ensure that a variety of user types could interact with the content and find it engaging. A recent cluster analysis of Pharmacy5in5 users’ engagement showed that pharmacy students were more engaged with the platform than pharmacists in practice [19]. Another challenge identified was the low quiz completion rate among users. Based on data tracking when users start a module, how and when they complete it, around 50% of users complete an entire module. To promote completion of the full module, we aimed to test the addition of a social giving feature where users earn charitable rewards for completing quizzes and modules. The charitable reward chosen for this study was donation of meals to Food Banks Canada.

The research on the most effective gamification features is still evolving [20], and the lack of high quality and well-grounded evidence due to the limited number of randomized controlled studies reported [21, 22]. Therefore, we conducted this study to explore whether the addition of a social reward feature to the platform would make it more engaging for users. The aim of this project is to pilot a social reward feature The objectives of the project are: 1) to assess the feasibility and acceptability of both the social reward feature and the study design; and 2) to provide preliminary estimates of the impact of social reward on users’ engagement.

## Methods

To assess the feasibility and acceptability of the social reward feature, a mixed-methods approach was used, combining a randomized controlled trial (RCT) with surveys and telephone interviews to provide a more in-depth understanding of user experience.

### Study design

**Fig. 1:**
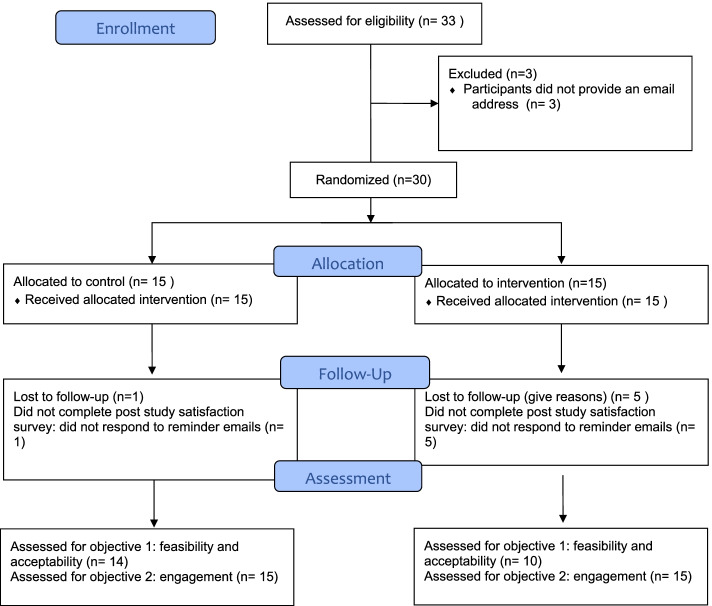
Study flowchart

A four-month, two-arm, web-based intervention, randomized controlled trial (RCT) was used to assess the effect of a social reward on pharmacy professionals’ engagement with the computer-based education platform Pharmacy5in5. The study was conducted between April 2021 and August 2021. The RCT was conducted in accordance with CONSORT-EHEALTH checklist [23], and CONSORT 2010 statement, extension to randomised pilot and feasibility trials [24]. Participants were randomly allocated to either the intervention group or the control group, as shown in the study flowchart below (Fig. 1). The assessment was conducted after four months.

### Recruitment

All registered users of Pharmacy5in5 located in the Canadian province of Ontario (n ≈ 9500) were invited to participate in the study via email. In the email, users received a consent form and a letter explaining the purpose of the study along with a link to a survey to provide their demographic details and email addresses. A total of two recruitment emails were sent to increase response rate [25, 26].

### Study procedure

Participants who completed the consent form were assigned a unique study identifier and randomly allocated to the intervention or the control group. Participants allocated to the intervention group had a social reward, while participants allocated to the control group did not have a social reward. Both groups had normal access to the Pharmacy5in5 platform and were asked to use the website as per their usual practice for a period of four months. Participants in the intervention group received a second email to notify them that they would “earn” one meal for donation every time they complete a quiz (with a minimum score of three out of five). They also received a weekly summary report of their total donations via email. Conversely, participants in the control group did not receive any additional updates. Throughout the study duration, participants were not prompted via email or any other method to complete the modules or quizzes to allow for assessment of any effect of the social reward on engagement. A logbook was kept throughout the study to keep a record of feasibility and acceptability indicators for study design.

After two months, the completion rate for quizzes was lower than expected among users, therefore the protocol was amended to conduct a mid-point survey to collect participants’ views on the social reward feature. Participants in the intervention group were invited via email to complete a five-minute mid-point-survey to assess the acceptability of the social reward intervention. The survey included two open-ended questions assessing what users liked about the social reward feature and what they would like to change.

At the end of the 4-month intervention period, both groups were invited via email to complete a 5–10 min 18-item satisfaction survey to assess acceptability of the social reward feature and the study design. The first section of the survey focused on user satisfaction with the type and amount of donations, their willingness to complete more modules with social reward features, and areas where the social reward can be improved. The second section assessed contamination bias and whether users knew or discussed their study group assignment with other users. At the end of the satisfaction survey, participants in the intervention group were asked if they were willing to participate in a telephone interview. Reminder emails were sent out to participants along with a link to the survey after 10 days, and again three weeks after the initial invitation to improve the response rate. Participants in the control group were only asked about contamination bias.

In the interviews, participants were asked about their experience with the social reward feature. Before the interview, the researcher explained to the participant the goal of the study, the main questions that the study will address, the questions that the participant will be asked, and the participant’s right to withdraw from the study. The telephone interview was scheduled at a time and location that was convenient for the participants and was audio recorded after receiving the participant's permission. The recordings were transcribed and anonymized before analysis.

To assess user engagement with the platform throughout the 16-week study duration, response data were generated on a weekly basis and included: a unique user ID for each user, quiz name, question title for each quiz, whether the question answer was correct (reported as true/false), module name, and time answer created. A chart was created using an Excel spreadsheet to extract key data from weekly response data, and included: number of quizzes attempted, number of modules attempted, number of modules completed in full, number of quizzes with a score of three out of five and number of meals donated.

### Instrument development

#### Satisfaction survey development

The quantitative study was conducted using an 18-item self-reported survey (see Additional file 1). Six statements were adapted from a satisfaction survey designed by Pelayo et al. to assess physician satisfaction with a computer-based education platform. [27] Two statements were added to address the implementation and maintenance of the social reward feature based on the RE-AIM framework (Reach, Effectiveness, Adoption, Implementation, and Maintenance) which is a valuable tool to assess implementation [28, 29]. Participant responses were assessed using a five-point Likert scale (1 = extremely likely; 5 = extremely unlikely). Open-ended questions were used to assess feasibility and acceptability of the social reward and the weekly donation reports. To validate the survey, the first draft was shared with four pharmacists and one pharmacy technician to assess the clarity and comprehension of the questions. Minor modifications were made to six questions and one new question was added to assess user interest in sharing news of their donation via social media. Next, the survey was piloted by five practicing pharmacists and three questions were modified based on these pharmacists’ comments.

#### Semi-structured interview guide development

The semi-structured interview guide was also developed to assess satisfaction and acceptability of the social reward feature. Specifically, how the food donation was received as the social reward for this study. It included a list of prompts to allow interview participants to share their insights on if or how the food donation motivated them to complete more quizzes and how the COVID-19 pandemic may have affected their engagement with the food donation. The guide was refined based on the results of the mid-point and satisfaction surveys. Examples of interview questions include: How do you think the food bank donation impacted your motivation to do quizzes/modules on Pharmacy 5in5? What is it about the food bank donation that you liked/disliked the most?

### Intervention

Pharmacy5in5 is a computer-based education intervention that aims to accelerate the adoption of best practices by pharmacy professionals. Pharmacy5in5 is designed to regularly release modules that cover five take home messages about a clinical or pharmacy practice topic. Each module has the following design components:

- One fast facts quiz with immediate feedback
- Six case-based quizzes, with delayed feedback [18, 19]
- Peer comparison
- Self-reflection questions to self-report behaviours
- Multimedia resources including short videos, infographics and flashcards.

**Fig. 2:**
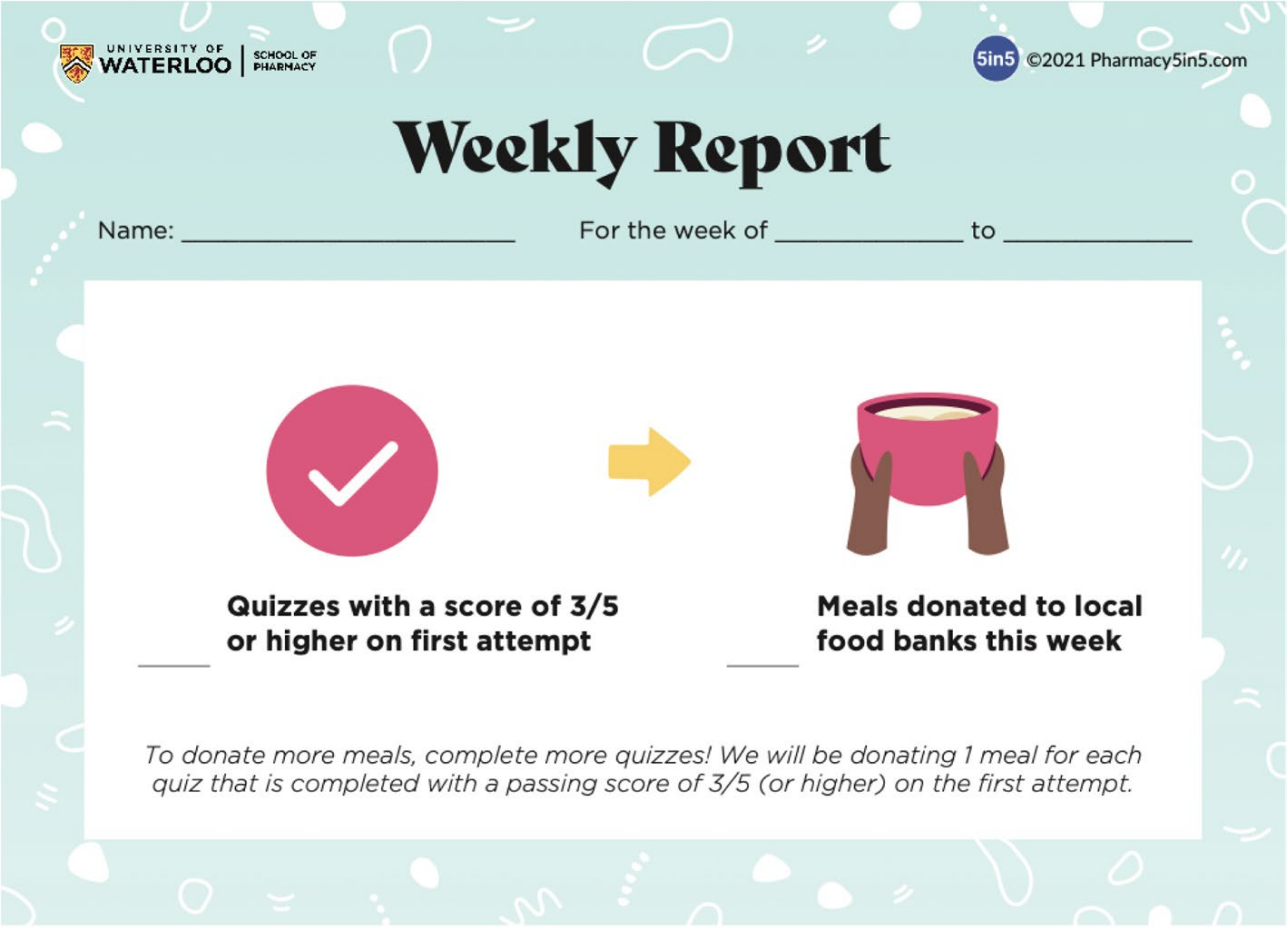
Weekly report template

For this study, a social reward feature was added to the platform where users can earn one meal as a reward for each quiz they complete with a passing score of at least three out of five. In addition, weekly donation reports were sent to users in the intervention group to reflect the total number of quizzes with passing scores and total number of meals donated each week. See Fig. 2 for the weekly report template.

### Outcome measure

#### Primary outcomes

The primary outcome measures of this study are the feasibility and acceptability of both the social reward feature such as mode of delivery and acceptability and the study design such as recruitment; retention and follow-up assessments. See Additional files 1 and 2 for the complete list of feasibility and acceptability indicators.

#### Secondary outcomes

The trial provided preliminary estimates of the impact of the intervention on user engagement defined as the number of quizzes completed by users, the number of modules completed in full by users (out of a possible seven quizzes per module) and the number of modules attempted (out of a possible 30 modules), where at least one quiz was completed (with a minimum score of 3/5). Any instances where users re-took a quiz were excluded.

#### Sample size calculation

As the aim of this study was to assess the feasibility of a larger trial to assess impact of the intervention on user engagement, no formal sample size calculation was undertaken. A total of 30 users with 15 in each arm were recruited [30, 31].

#### Randomization and blinding

Participants were randomized in a 1:1 allocation ratio using a computer-generated random number. Given the nature of the study, it was not feasible to blind the participants. However, only the principal investigator was aware of the allocations. The study team was blinded to allocation until the analysis was complete.

### Data analysis

Using data automatically generated by the Pharmacy5in5 platform, the outcome measures were compared across the two groups using descriptive statistics. User engagement assessed using number of modules completed, number of quizzes completed, number of modules attempted and number of quizzes with a score of at least three out of five were compared between the intervention and control groups using independent t-test.

The quantitative survey results were analyzed using descriptive statistics. Cronbach's alpha was used to assess reliability of the survey and the value was 0.6. Qualitative data analysis was guided by the Framework Method outlined by Gale et al., [32] which includes familiarization, coding, indexing, charting, and interpretation of data. An inductive analysis was used to code the transcripts to capture any emerging themes. Once all data were coded, an Excel spreadsheet was used to generate a matrix that included participant responses with matching codes. Finally, the data was interpreted by reviewing the matrix and exploring connections in participants response to help provide explanations of trends in data and generate themes. All generated themes were reviewed by the research team for agreement. Chi-square and Fisher’s exact test were used to assessing differences in baseline demographic between the control and intervention groups.

## Results

### Feasibility and acceptability of the trial design

A total of 9250 users were invited to participate. As a measure of feasibility, time needed to reach sample size was six days after sending two reminder emails, all users signed consent forms and completed the demographic survey (*n* = 30; 100%). All users agreed to be randomized with 15 users in each arm. As per Table 1, most respondents were female (81%), received their training in Canada (75%), held a bachelor’s degree (54.6%), and were practising in community pharmacies (70%). There were no statistically significant differences between the demographic characteristics of the two study groups.

**Table 1:** Demographics of the control group and intervention group participants

Demographics	Intervention group (*n* = 15)	Control group (*n* = 15)
**Years of pharmacy practice experience**		
Less than 1 year	3	0
1–5 years	3	4
6–10 years	2	4
11–20 years	4	3
More than 20 years	3	4
**Gender**		
Woman	13	11
Man	2	4
**Location of training**		
Canada	11	12
In the United States	1	0
Outside North America	3	3
**Highest level of education**		
Bachelor	9	8
Entry-level PharmD	3	1
Masters	1	1
Postgraduate PharmD	1	2
Pharmacy student		1
Pharmacy technicians	1	2
**Primary site of practice**		
Community: Independent pharmacy	4	6
Community: chain or franchise	8	3
Hospital in-patient	1	5
Other	2	1

In terms of feasibility, a total of seven users (47%) in the intervention group completed the mid-point survey and 10 users (67%) completed the satisfaction survey at the end of the study. A total of four survey respondents in the intervention group were interested in the telephone interviews. All were contacted, however, only two users agreed to proceed with the telephone interviews. In the control group, 14 users (93%) completed the satisfaction survey.

In terms of contamination, all the users who completed the survey reported that they did not know other users who participated in the study, did not discuss their group allocation with a colleague, nor knew whether their colleagues were assigned to a different group.

### Feasibility and acceptability of the social giving feature

Throughout the 16-week study duration, 225 out of 240 weekly reports were sent via email on time (94%), and 15 weekly reports were delayed by one day (6%). Out of 15 users in the intervention group, 13 users received weekly report with the correct number of meals throughout the 16 weeks study duration. Only two users received one weekly report with incorrect number of meals.

In the mid-point survey, most users rated the social reward feature as highly acceptable. Many noted that they liked how this intervention helped people in need while users completed educational modules. In terms of changes to the food donation feature, one pharmacist suggested that the weekly report should be sent along with a reminder to complete modules.

*“I am wondering if the timing of the email would be more effective if it was sent on the following Wednesday with a reminder of completing a module or two by the end of the week.” Participant 2*

Another pharmacist suggested to make the donations more automatic. Another comment was regarding the difficulty of re-taking modules and quizzes that were already completed before the addition of the feature to the platform.

In the satisfaction survey, most of the users were highly satisfied with the intervention, with 8/10 (80%) reporting they were extremely or somewhat satisfied with the overall experience with donating meals as a reward, 9/10 (90%) were extremely or somewhat satisfied with the amount of one meal per quiz and 9/10 (90%) were extremely or somewhat satisfied with the weekly donation report. Only 5 out of 10 users reported that they would recommend a module with charitable rewards feature to a friend or colleague. A total of 80% reported that they would be more likely to start a module with a charitable rewards feature, and 80% reported that they would be more likely to finish a module with a charitable rewards feature.

Regarding the dollar amount of donation, six out of the 10 users reported that a higher donation amount would not motivate them more to complete more quizzes. Among the four users who agreed that a higher donation amount would motivate them more, they suggested a minimum of $5 or three meals for each quiz rather than one meal. Other suggested charitable donations that might be more motivating than food banks such as women’s shelters, clothes, and education supplies to those in need.

In terms of acceptability of the weekly report, six out of ten users preferred to receive their report on monthly basis. Most of the users (9/10) preferred to have a donation report history available on their Pharmacy5in5 account. Six out of ten would like to see their donations acknowledged as a badge for each donation after each quiz. Surprisingly, six out of ten users felt they were unlikely to share earned donations via social media. Moreover, weekly report fidelity was high with all users reporting that they received their report consistently each week and thinking the total amount of donations accurately reflected the number of quizzes successfully completed.

In the interviews, users discussed how they felt more motivated to complete more quizzes with the meals donating feature. They also shared that the weekly reports were good reminders to complete more quizzes.

*“The weekly report was a good reminder when it showed up in my inbox, I was like, "Oh yeah, I haven't done anything of Pharmacy5in5 quizzes lately... and having the donations was just like an added reminder to do stuff.” Participant 2*

Interviewed participants expressed concerns about sustainability and source of funding with a higher donation amount:

*"For me, the fact that even that it was one meal, that was my driver. If it was three meals, I don't think I would've found more time...****The thing is that where's that funding coming from****? Because you're really learning for your own personal benefit. So, I would have a hesitancy to put much more value on that [ 5in5 quizzes] other than [one meal]” Participant 1*

*“I think one meal was pretty standard. Because the quizzes are quick, right? I feel like it would be excessive to have more than one meal, especially just because they're so quick to finish…I guess a higher donation amount would be good, but then ****I would fear that it would not be sustainable for who was providing the money****.” Participant 2*

The two interviewed participants also mentioned a number of suggested changes to make the social reward feature more motivating including setting weekly goals for the number of meals to be donated and to be given the choice to select their preferred donation. They also highlighted that they prefer the weekly report to include further information about the number of meals donated by other users to get them more motivated.

### Potential impact of the intervention on user engagement

The total number of quizzes completed in full by users in the intervention group was 267, while the total number of quizzes completed in full by users in the control group was 97, as shown in Table 2. The number of quizzes with a score of at least three out of five completed by users in the intervention group was approximately three times higher than the number completed by users in the control group (*n* = 250 quizzes Vs. *n* = 80 quizzes p-value 0.0131). Overall, 250 meals were donated to Food Banks Canada throughout the study duration.

**Table 2:** Users engagement by the study groups

User engagement elements	Control group (*n* = 15)	Intervention group (*n* = 15)	*P* value*
Number of quizzes completed in full	97	267	0.023
Number of modules attempted	28	81	0.013
Number of modules completed in full	10	34	0.0157
Number of quizzes with a score of at least 3 out of 5	80	250	0.0131

^*^Independent t-test

Overall, 13 out of 15 (86.6%) participants in the intervention group completed at least one quiz with a score of at least three out of five and donated at least 1 meal throughout the study duration. Only two participants in the intervention group did not donate any meals or complete any quizzes throughout the study duration. In the control group, 8 out of 15 (53.3%) users completed at least one quiz with a score of at least three out five throughout the study duration.

**Fig. 3:**
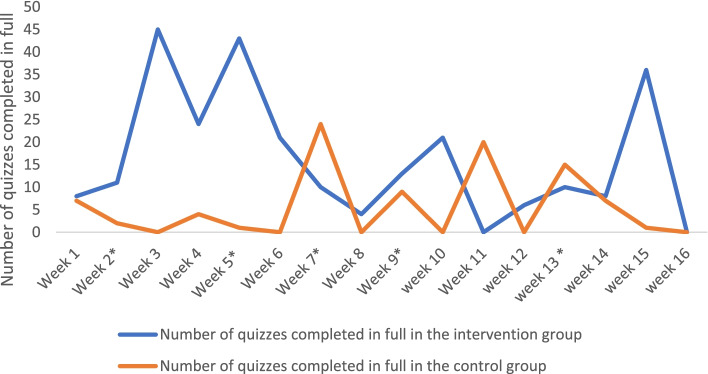
Number of quizzes completed in full by study participants throughout the study duration. _* A week with a new module/quiz released_

Figure 3 highlights the number of quizzes completed in full by users in the two study groups during the 16-week study duration. In the first week both groups showed similar level of engagement with the platform. Intervention group engagement with the platform started to increase in the second week with more quizzes completed and reached a peak in the third and fifth week of the study. Engagement dropped in the second and third months of the study expect for a peak in week 10. Number of quizzes started to increase again in the fourth month and peaked in week 15.

Overall, in the first two months of the study, users in the intervention group completed quizzes regardless of whether there was a new quiz/module released or not. However, in the last two months they seemed more engaged when a new module/quiz was released. Users in the control group had a lower level of engagement overall, and tended to complete more quizzes in weeks where a new module/quiz released. A total of five new quizzes were released throughout the four-month study duration. Of note, quizzes were focused on COVID-19 and vaccination content. In terms of content, both groups completed quizzes related to COVID-19 as well as other pharmacy-related topics.

## Discussion

In this study a pilot trial was conducted to assess a social reward feature with pharmacy professionals. The study demonstrated the feasibility and acceptability of the social reward feature, where most users were highly satisfied with the food donation, and the weekly reports. Moreover, the study demonstrated the feasibility of the study design, where users were successfully recruited and randomized and followed over a period for four months with no contamination. However, there were challenges with retention. In terms of potential impact of the social reward on users’ engagement, the study showed that users in the intervention group were more engaged with the platform compared to users in the control group.

With regards to limitations, the low response rate to the survey and the telephone interviews among users in the intervention group should be noted. This study was conducted in a period when the bulk of COVID-19 vaccination was conducted in Ontario pharmacies, which may have limited the availability and willingness of pharmacists and pharmacy technicians to participate in the survey and interviews. Other studies have recommended to use rewards to improve retention rate in a full scale RCT [33, 34]. While a significant difference can be seen in the numbers despite the small number of participants, a larger sample size is needed for the mixed methods to reach saturation. Another limitation is recall bias with the survey responses regarding questions assessing the weekly reports. Selection bias is another potential limitation, as only pharmacy professionals interested agreed to participate [35]. One important challenge with this social giving element is securing funding for sustainability. Potential sources of funding are pharmacy regulatory bodies, interested employers and access fees to be paid by users.

The study highlighted rewards as a promising game element that can improve pharmacy professionals’ engagement with a computer-based education platform by incenting the completion of more quizzes and attempting more modules. However, the reward did not sustain the engagement long term, as users became less engaged after two months. This is in line with results of previous studies in education and marketing which suggest that the positive effect of gamification elements such as rewards may wear off with time, due to the novelty effect [22, 36, 37]. Another explanation is that rewards enhance extrinsic rather than intrinsic motivation leading to an immediate and short-term impact on engagement [38].

Another key finding was the positive effect of providing updated and relevant content on engaging pharmacy professionals with the platform. Users in both groups were more engaged with the platform when new COVID-19-related content was released. Research has shown that a platform is perceived to be highly engaging when the content is relevant and appealing. However, in order to sustain engagement among pharmacy professionals, a platform must provide updated content and ongoing learning opportunities [7].

## Conclusion

This pilot study demonstrates the feasibility of conducting a web-based RCT with high fidelity and acceptability by pharmacy professionals. The study also demonstrates high satisfaction with a social reward intervention. Moreover, preliminary estimates suggest that a social giving feature can improve pharmacy professionals’ engagement with a computer-based education platform over the short term. The study also showed that it’s feasible to quantify engagement using the number of quizzes and modules attempted and completed on a computer-based education platform. Further efforts should be made to enhance pharmacy professionals’ retention and follow-up assessment in a full-scale RCT.

## Supplementary Information

**Additional file 1.** Feasibilityindicators of the social reward and the study design [28, 29]. 

**Additional file 2.** Acceptabilityindicators of the social reward and the study design.

## Data Availability

The datasets used and/or analysed during the current study are available from the corresponding author upon reasonable request.
