# Supplementary material for: Effectiveness of social giving on the engagement of pharmacy professionals with a computer-based education platform: a pilot randomized controlled trial
Source: BMC Med Educ. 2022 Apr 7;22:253. doi: 10.1186/s12909-022-03310-0 (PMC8988535; doi:10.1186/s12909-022-03310-0)
Supplement: Supplementary file 1 — Additional file 1. Feasibilityindicators of the social reward and the study design [28, 29]. [file 12909_2022_3310_MOESM1_ESM.docx]

Additional file 1: Feasibility indicators of the social reward and the study design^28,29^

| The social reward | Element | Question | Indicator | Assessment tools |
| --- | --- | --- | --- | --- |
|  | Dose of the intervention | Is the planned dose of weekly report feasible? | % of reports delivered as intended  # of users received reports as intended | Logbook |
|  | Mode of delivery | Is the planned mode of delivery of weekly report via email feasible? | Intervention researchers’ opinion regarding feasibility |  |
| The study design | Recruitment | What is the number of users that were recruited compared to the number of users that were evaluated for eligibility? | Number of recruited users/users evaluated for eligibility. | Logbook |
|  |  | What are the characteristics of participating users? | Sociodemographic data. | Logbook |
|  |  | How much time is needed to reach sample size? | Time needed to reach sample size. | Logbook |
|  | Randomization | Is the method of randomization feasible? | Number of users in study groups | Logbook |
|  | Allocation concealment | Is the method of allocation concealment feasible? |  |  |
|  | Measure methods | Is it feasible to use 5i5n data to measure engagement? | Number of quizzes and modules attempted | Platform database |
|  | Data collection | Is it feasible to perform data collection as planned? | % of data collection performed as planned. |  |
|  | Retention** | Are we able to retain all users throughout the study? | Number of lost to follow-up and reason. | logbook |
|  | Contamination | Is there a risk of contamination? (participants in either group receive the intervention intended for  those in the other group) | % of users that were exposed to the content of the intervention.  **no more than 5% of users received the intervention intended for  those in the other group ^30^ | - Log book  - Survey |
|  | Partial disclosure** | Is it feasible to perform partial disclosure as planned? | Number of users agreed with and signed the post-debriefing consent form | post-debriefing consent form |
