# Supplementary material for: Effectiveness of social giving on the engagement of pharmacy professionals with a computer-based education platform: a pilot randomized controlled trial
Source: BMC Med Educ. 2022 Apr 7;22:253. doi: 10.1186/s12909-022-03310-0 (PMC8988535; doi:10.1186/s12909-022-03310-0)
Supplement: Supplementary file 2 — Additional file 2. Acceptabilityindicators of the social reward and the study design. [file 12909_2022_3310_MOESM2_ESM.docx]

Additional file 2: Acceptability indicators of the social reward and the study design

| The social reward | Element | Question | Indicator | Assessment tools |
| --- | --- | --- | --- | --- |
|  | Dose of the intervention | Is the planned dose of weekly report appropriate? | Preferences of users in terms of the dose delivered. | Survey/Interviews |
|  |  | Is the planned monetary amount of social giving appropriate? | Preferences of users in terms of the monetary amount | Survey/Interviews |
|  | Mode of delivery | Is the planned mode of delivery for weekly report appropriate? Via email | Preferences of users in terms of the mode of delivery | Survey/Interviews |
|  | General acceptability of the intervention | Satisfaction with the social reward |  | Survey/Interviews |
| The study design | Randomisation | Do users accept to be randomized? | Number of users agreed to be randomized/ asking to crossover  **no more than 5% of all recruited subjects asked to be crossed over from one modality to the other^30^ | Logbook |
|  | Data collection | Is it acceptable for users to complete surveys? | 80% of users accept. | Logbook |
|  |  | Is it acceptable for users to complete an interview? | 80% of users accept. | Logbook |
